# The Interaction Between Long Non-coding RNA HULC and MicroRNA-622 via Transfer by Extracellular Vesicles Regulates Cell Invasion and Migration in Human Pancreatic Cancer

**DOI:** 10.3389/fonc.2020.01013

**Published:** 2020-06-23

**Authors:** Kenji Takahashi, Kazuya Koyama, Yu Ota, Hidetaka Iwamoto, Keisuke Yamakita, Satoshi Fujii, Yohei Kitano

**Affiliations:** ^1^Division of Metabolism and Biosystemic Science, Department of Medicine, Asahikawa Medical University, Asahikawa, Japan; ^2^Department of Laboratory Medicine, Asahikawa Medical University, Asahikawa, Japan

**Keywords:** long non-coding RNA, microRNA, epithelial-mesenchymal transition, invasion, migration, pancreatic ductal adenocarcinoma

## Abstract

Although non-coding RNAs (ncRNAs) are involved in disease pathogenesis, their contributions to pancreatic ductal adenocarcinoma (PDAC) remain unclear. Recently, the interrelationship between two classes of ncRNA, long non-coding RNAs (lncRNAs), and microRNAs (miRNAs), has been reported to contribute to the epigenetic regulation of gene expression in several diseases including cancers. Moreover, some ncRNAs can be transferred by extracellular vesicles (EVs) from their donor cells to recipient cells. We previously verified that lncRNA HULC is up-regulated in PDAC cells and the intercellular transfer of HULC by EVs can promote PDAC cell invasion and migration through the induction of epithelial–mesenchymal transition (EMT). Therefore, we identified the miRNA that could target HULC and investigated the functional contributions of the miRNA–HULC interaction and EV transfer of miRNA to the EMT pathway in PDAC. Microarray analysis revealed 187 miRNAs that were decreased to <0.87-fold in Panc-1 cells treated with TGF-β compared with the control. Of these, miR-622 was predicted to target HULC directly by bioinformatics analysis. Expression of miR-622 was significantly down-regulated by TGF-β in a panel of PDAC cells. miR-622 overexpression by a miRNA mimic significantly decreased HULC expression, increased E-cadherin expression, and decreased expression of Snail, N-cadherin, and vimentin. Moreover, overexpression of miR-622 significantly reduced cell invasion and migration whereas inhibition of miR-622 increased HULC expression and promoted EMT signaling, invasion, and migration of PDAC cells. Furthermore, incubation with miR-622-overexpressing EVs could transfer miR-622, which significantly elevated miR-622 expression and decreased cell invasion and migration via inhibition of the EMT pathway in recipient PDAC cells. These results provide mechanistic insights into the development of PDAC by demonstrating that miR-622, as a miRNA downregulated by TGF-β, could target HULC and suppress invasion and migration by inhibiting EMT signaling via EV transfer. These observations may identify EV-encapsulated miRNA as a novel therapeutic target for human PDAC.

## Introduction

Pancreatic ductal adenocarcinoma (PDAC) is a highly invasive and metastatic cancer, and more than 80% of patients have an advanced stage at diagnosis. Invasion or metastasis at the early stage of PDAC are associated with poor prognosis (1, 2). The epithelial–mesenchymal transition (EMT) process plays important roles in the differentiation of multiple tissues and organs through morphological transformation from epithelial cells into cells with a mesenchymal phenotype, and is thought to be an crucial trigger for tumor invasion and migration in PDAC (3, 4). Long non-coding RNAs (lncRNAs), which are non-protein-coding RNAs (ncRNAs) >200 nucleotides in length, are being increasingly elucidated to regulate many biological processes via diverse mechanisms (5). Although de-regulated expression of lncRNAs has been reported to be related to several diseases (6, 7), their roles in EMT in PDAC are not well-understood. We recently showed that the lncRNA highly up-regulated in liver cancer (HULC) promoted PDAC cell invasion and migration via induction of the EMT pathway (8). However, there is little information about the roles of lncRNAs in PDAC development.

The regulatory mechanisms of interactions between lncRNAs and microRNAs (miRNAs) reportedly affect epigenetic regulation in several diseases (9). The crosstalk between these ncRNAs occurs by competing for shared miRNA response elements, suggesting that lncRNAs can interact with specific miRNAs and work as competing endogenous RNAs (ceRNAs) to inhibit degradation of the targeted transcripts of those miRNAs (10). The regulation by ceRNA is exemplified by the lncRNA differentiation antagonizing non-protein coding RNA, which binds miR-33a-5p and regulates the expression level of its downstream miRNA-target genes in esophageal squamous cell carcinoma (11). The lncRNA X-inactive specific transcript functions as a ceRNA of miR-126 and then regulates the IRS1/PI3K/Akt pathway in glioblastoma cells (12). Meanwhile, we recently reported that miR-133b could target lncRNA HULC and inhibit the EMT pathway via suppression of HULC expression in PDAC cells, suggesting that lncRNAs can be targeted by miRNAs (8). Based on this knowledge, we aimed to identify other interactions between HULC and miRNAs that could modulate tumor cell invasion and migration via EMT regulation.

Extracellular vesicles (EVs) exhibit a diverse range of sizes (100~5,000 nm in diameter) and are generally known in the literature as exosomes, microvesicles, or apoptotic bodies. EVs enclose cytosolic proteins, lipids, and nucleic acids (13, 14). EVs can transfer their contents from donor cells to recipient cells and mediate recipient cell phenotypes. In addition to proteins, lipids, and mRNAs, miRNAs and other ncRNAs are also possible EV cargoes. The theory that ncRNAs contained within EVs can be delivered to recipient cells and directly modulate their targeted mRNAs is widely recognized as an actively explored hypothesis in several cancer fields. This mechanism of EV-mediated intercellular communication has been revealed by several authors (13, 15–17). We previously reported that miR-30e could be transferred by cholangiocarcinoma (CCA) cell-derived EVs, and suppress recipient CCA cell invasion and migration (18). In PDAC, some investigators reported that EV-encapsulated miRNAs, such as miR-196a, miR-1246, or miR-4644, could contribute to tumor development and play crucial roles in the diagnosis as tools of liquid biopsy (19–21). Therefore, we focused on identifying the mechanisms of ncRNA transfer by PDAC-derived EVs and investigated their contributions to PDAC development.

In the present study, we identified a miRNA that can negatively regulate HULC and be involved in the EMT pathway. Moreover, we investigated the contributions of the interrelationship between HULC and the miRNA to PDAC cell invasion and migration. Furthermore, we elucidated the regulatory mechanisms of PDAC cell invasion and migration via the intercellular miRNA transfer by EVs.

## Materials and Methods

### Cell Lines and Reagents

The human pancreatic cancer cell lines Panc-1, MIA PaCa-2, and BxPC-3 were obtained from the American Type Culture Collection (ATCC; Manassas, VA, USA), and KP-3 and QGP-1 were purchased from the Japanese Collection of Research Bioresources Cell Bank (Osaka, Japan). Panc-1 cells were cultured in high-glucose Dulbecco's modified Eagle's medium (DMEM; Thermo Fisher Scientific, Waltham, MA, USA) containing 10% fetal bovine serum (FBS) and 1% penicillin–streptomycin (Thermo Fisher Scientific). MIA PaCa-2 cells were cultured in high-glucose DMEM containing 10% FBS, 2.5% horse serum, and 1% penicillin–streptomycin. BxPC-3, KP-3, and QGP-1 cells were cultured in Roswell Park Memorial Institute (RPMI) 1640 medium (Thermo Fisher Scientific) containing 10% FBS and 1% penicillin–streptomycin. All cells were cultured at 37°C in an atmosphere containing 5% CO_2_. TGF-β (TGF-β1) was obtained from EMD Millipore (Billerica, MA, USA). Cells were treated with 10 ng/mL TGF-β for 72 h to induce EMT.

### Isolation of EVs

Vesicle-depleted medium prepared using exosome-depleted FBS (Thermo Fisher Scientific) was used for all studies. Isolation of EV was performed as described previously (8). The ultracentrifuged pellet comprised an EV preparation that contained a heterogenous population of EVs and was used to isolate EV RNA or other studies, or was resuspended in 50–100 μL of phosphate-buffered saline and stored at −80°C. The protein yield was evaluated using a bicinchoninic acid (BCA) protein assay kit (Thermo Fisher Scientific), and EV morphology was visualized by transmission electron microscopy.

### Isolation of RNA

Total RNA was extracted from cells using the miRNeasy Mini Kit (Qiagen, Valencia, CA, USA), and extracellular RNA (exRNA), including EV RNA, was extracted using ExoQuick-TC (System Biosciences, Mountain View, CA, USA) in accordance with the manufacturer's instructions. For the latter, cells (1 × 10^6^) were seeded in 11 mL of EV-depleted medium on 10-cm dishes. After 72–96 h, the medium was collected and sequentially centrifuged at 3,000 × *g* for 15 min to remove cells and cell debris. Next, 10 mL of supernatant was transferred to a sterile vessel and combined with 2 mL of ExoQuick-TC. After an overnight precipitation at 4°C, exRNA was extracted using the SeraMir Exosome RNA Amplification Kit (System Biosciences) in accordance with the manufacturer's instructions. RNA concentration was measured using a NanoDrop ND-1000 instrument (NanoDrop Technologies, Wilmington, DE, USA).

### miRNA Microarray Analysis

We referred to and used the data from a previous miRNA microarray analysis (8). The miRNA microarray data can be accessed using the National Center for Biotechnology Information GEO Database (https://www.ncbi.nlm.nih.gov/geo/query/acc.cgi) under accession no. GSE121369.

### Polymerase Chain Reaction (PCR) Analysis

RNA was treated with RNase-free DNase I (Qiagen) and reverse-transcribed to cDNA using the iScript cDNA Synthesis Kit (Bio-Rad Laboratories, Hercules, CA, USA). Real-time quantitative reverse-transcription PCR (qRT-PCR) was performed to analyze mRNAs using an Applied Biosystems 7300 Real-Time PCR System (Applied Biosystems, Foster City, CA, USA), and expression was normalized to that of RNU6B (U6) with SYBR green I (SYBR Advantage qPCR Premix; Clontech Laboratories, Mountain View, CA, USA). The following PCR primers were used: E-cadherin forward: 5′-TGCACCAACCCTCATGAGTG-3′ and reverse: 5′-GTCAGTATCAGCCGCTTTCAG-3′; Snail forward: 5′-TTCTCACTGCCATGGAATTCC-3′ and reverse: 5′-GCAGAGGACACAGAACCAGAAA-3′; N-cadherin forward: 5′-TCGCCATCCAGACCGACCCA-3′ and reverse: 5′-TGAGGCGGGTGCTGAATTCCC-3′; vimentin forward: 5′-CCTGAACCTGAGGGAAACTAA-3′ and reverse: 5′-GCAGAAAGGCACTTGAAAGC-3′; U6 forward: 5′-CTCGCTTCGGCAGCACA-3′ and reverse: 5′-AACGCTTCACGAATTTGCGT-3′. Expression of mature miRNA-622 was assessed using the TaqMan Human MicroRNA Assay Kit (Applied Biosystems) and normalized to the expression of U6.

### Transfection of miRNA Mimic or Inhibitor

PDAC cells were transfected with a mirVana miR-622 mimic, inhibitor, or negative control (Applied Biosystems) using Lipofectamine RNAiMAX (Life Technologies, Grand Island, NY, USA). After 48–72 h, the cells were collected and used for further experiments.

### Western Blot Analysis

Total protein was isolated from cultured cells using cOmplete Lysis-M EDTA-free and cOmplete, Mini, EDTA-free Protease Inhibitor Cocktail Tablets (Roche, Basel, Switzerland). Protein concentrations were assessed using a BCA protein assay kit (Thermo Fisher Scientific). Equivalent amounts of protein samples were mixed with NuPAGE LDS Sample Buffer (4×), separated in NuPAGE Novex 4–12% Bis-Tris Gels (Life Technologies), and transferred onto nitrocellulose membranes. The membranes were blocked with blocking buffer (TBS-T [25 mM Tris-HCl pH 7.4, 125 mM NaCl, 0.05% Tween-20] with 5% bovine serum albumin [Sigma-Aldrich, St. Louis, MO, USA]) for 1 h, and incubated overnight at 4°C with the following primary antibodies and subsequently with corresponding secondary antibodies for 1 h at room temperature. Antibodies and dilutions used were rabbit monoclonal anti-E-cadherin (1:1000, Cell Signaling Technology, Danvers, MA, USA), goat polyclonal anti-Snail (1:1,000; Abcam, Cambridge, UK), rabbit monoclonal anti-N-cadherin (1:1,000; Cell Signaling Technology), rabbit monoclonal anti-vimentin (1:1,000; Cell Signaling Technology), and mouse monoclonal anti-β-actin (1:5,000; Santa Cruz Biotechnology, Dallas, TX, USA). Immunoreactive bands were detected using the ChemiDoc XRS + Imaging System (Bio-Rad Laboratories), and protein levels are presented relative to that of β-actin.

### Cell Proliferation and Viability Assays

For the cell proliferation assay, cells (1 × 10^4^ per well) were passaged in 24-well plates. Trypan blue staining was done at the indicated time points, and the number of viable cells was shown relative to that at baseline. For the cell viability assay, cells (1 × 10^4^ per well) were plated into 96-well plates in appropriate media. Cell viability was assessed by CellTiter 96 AQueous One Solution Assay (Promega, Madison, WI, USA). Background correction was performed by subtracting the background fluorescence of wells without cells.

### Cell Invasion and Migration Assays

Transwell permeable support 24-well plates (Corning Inc., Corning, NY, USA) were used for cell invasion assays. The Transwell plates were pre-coated with pre-diluted Matrigel (5 μg/μL; BD Biosciences, Bedford, MA, USA). Cells (5 × 10^4^ per well) were seeded in 100 μL of serum-free medium in the upper chamber, and the lower chamber of the Transwell was filled with 600 μL of medium supplemented with 10% FBS. After a 24-h incubation, cells remaining on the upper surface of the Transwell chamber were removed using a cotton swab. The cells that had invaded through the Matrigel to the bottom of the insert were fixed and stained using Diff-Quik (Sysmex, Hyogo, Japan). The number of migrated cells was detected in three random high-powered fields under a light microscope, and the relative number was counted. Cells (4 × 10^5^ per well) were plated into 6-well plates and grown to 90% confluence for cell migration assays. Straight scratches were made using a sterile pipette tip. Floating cells were removed, and the cultures were maintained. Images of wounds were taken immediately and after 24 h. Cell migration was assessed by measuring the wound closure.

### Luciferase Reporter Assays

HULC, containing the miR-622 binding site sequence, was cloned into the firefly luciferase reporter vector. The vector and an empty vector were purchased from OriGene Technologies (Rockville, MD, USA). Cells (2 × 10^5^ per well) were plated into 6-well plates and treated with 12.5 nM miR-622 or a negative control mimic. After 24 h, those cells were co-transfected with 0.2 μg of firefly luciferase reporter vector containing the HULC sequence or the empty vector and transfected with 0.1 μg of *Renilla* luciferase reporter pRL-SV40 (Promega) using Lipofectamine 2000 (Thermo Fisher Scientific). After another 24 h, luciferase activity was evaluated using the Dual-Luciferase Reporter Assay (Promega). Relative firefly luciferase activity was normalized to *Renilla* luciferase activity.

### Statistical Analysis

Data are shown as the mean and standard error of at least three independent experiments, unless indicated otherwise. Comparisons between groups were performed using the two-tailed Student's *t*-test, one-way analysis of variance, or Mann–Whitney test, followed by the Bonferroni *post-hoc* test. Differences were considered statistically significant at *P* < 0.05.

## Results

### HULC Is an EMT Inducer of PDAC and a Direct Target of miR-622

**Figure 1:**
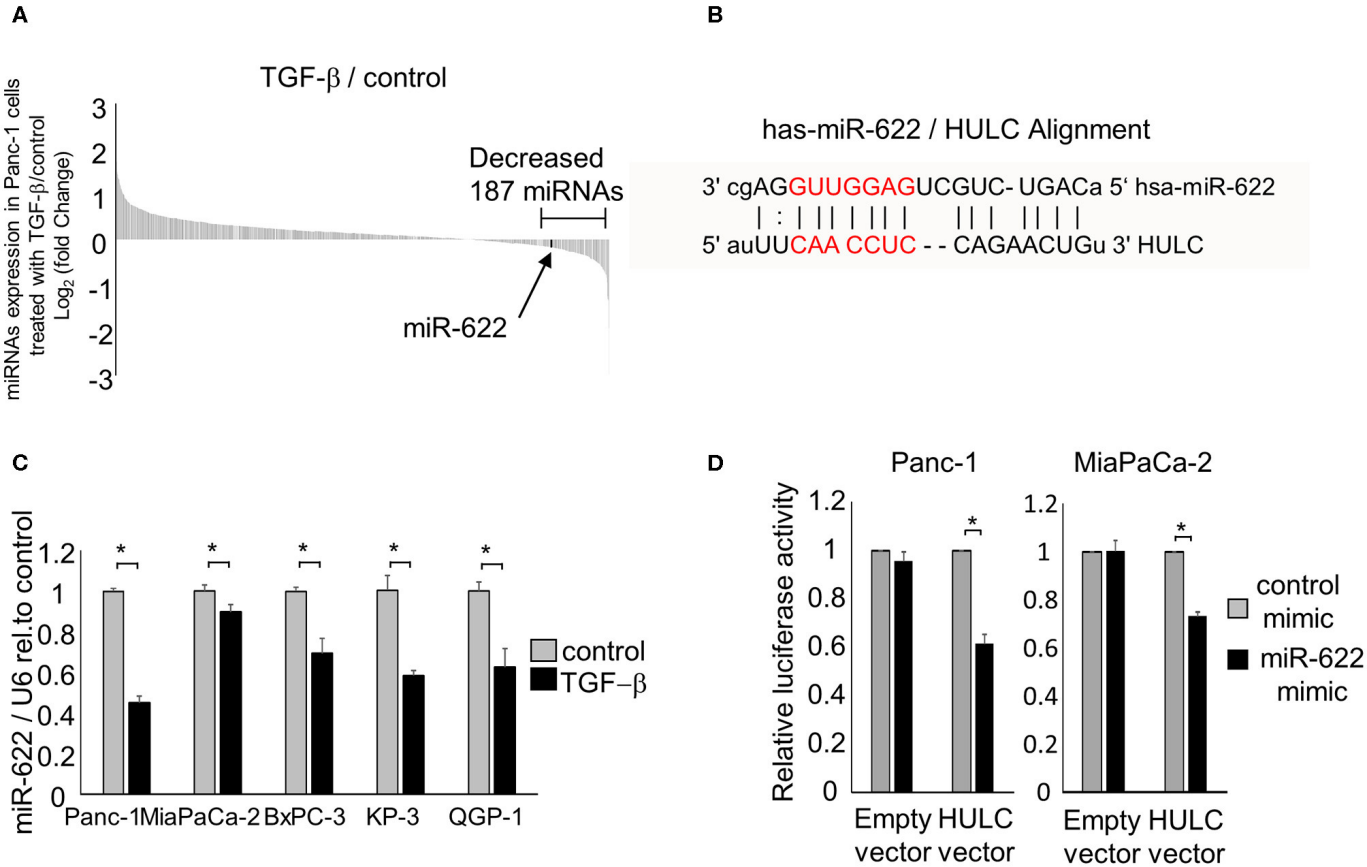
miRNAs that can target HULC in PDAC cells. **(A)** Panc-1 cells were treated with or without (control) 10 ng/mL of TGF-β. After incubation for 72 h, RNA was isolated, and expression profiling of 2,555 miRNAs was performed. The expression of 1,719 miRNAs was detected in Panc-1 cells. Waterfall plot of all the up- and down-regulated genes in TGF-β-treated Panc-1 cells compared to those of non-treated (control) cells is shown. Expression of 187 miRNAs was decreased <0.87-fold in Panc-1 cells treated with TGF-β. **(B)** miR-622 was predicted to target HULC directly by bioinformatics analysis on miRNA.org. **(C)** RNA was isolated and miR-622 expression was assessed by qRT-PCR in PDAC cells treated with 10 ng/mL TGF-β for 72 h. miR-622 expression relative to the controls is presented. Gene expression was normalized to that of U6. **(D)** Panc-1 and MIA PaCa-2 cells were transfected with 12.5 nM miR-622 or control mimic and, after 24 h, were co-transfected with 2.0 μg of the HULC firefly luciferase reporter vector or empty vector and 0.1 μg of the *Renilla* luciferase reporter pRL-SV40. Relative firefly luciferase activity was measured after a further 24 h and normalized to *Renilla* activity. Bars are the means ± SEM of three independent experiments. *P < 0.05.

It has been reported that TGF-β induces the EMT pathway, which triggers invasion and metastasis in PDAC. We recently reported that lncRNA HULC could promote tumor cell invasion and migration via induction of the EMT pathway in human PDAC cells. Also, HULC could be transferred by PDAC-derived EVs and modulate recipient PDAC cell phenotypes. Recently, the relationship between miRNAs and lncRNAs was reported to affect epigenetic regulation in digestive cancers (22). Therefore, to elucidate the regulatory mechanisms of EMT by targeting HULC, we aimed to investigate the mechanisms of HULC–miRNA interactions. We identified candidate miRNAs that can target HULC and are down-regulated by TGF-β (TGF-β1) using previous miRNA microarray data (8). We assumed that candidate tumor-suppressing miRNAs would inhibit EMT pathway through targeting HULC. The microarray analysis detected 187 miRNAs whose expression was reduced <0.87-fold in Panc-1 cells treated with TGF-β compared with the control (Figure 1A). Among them, miR-622 was predicted to target HULC directly based on bioinformatics analysis on miRNA.org (Figure 1B). miR-622 expression was down-regulated by TGF-β treatment in a panel of pancreatic cancer cells (Figure 1C). miRNAs are involved in the post-transcriptional regulation of RNA expression through their complementary binding to the 3′ untranslated region of their RNA targets (23). Bioinformatics analysis by miRNA.org revealed that HULC has a seven-nucleotide miR-622 binding site. Hence, to validate whether miR-622 can directly target HULC, we performed co-transfection of a miR-622 mimic and a firefly luciferase reporter vector including the target site of HULC into Panc-1 and MIA PaCa-2 cells. HULC firefly luciferase activity was significantly attenuated in miR-622-overexpressing cells (Figure 1D), confirming the specific interaction between miR-622 and HULC. According to these results, we focused on miR-622 as a candidate EMT-suppressing and onco-suppressive miRNA in further experiments.

### miR-622 Overexpression Inhibits Cell Invasion and Migration via Suppression of EMT by Targeting HULC

**Figure 2:**
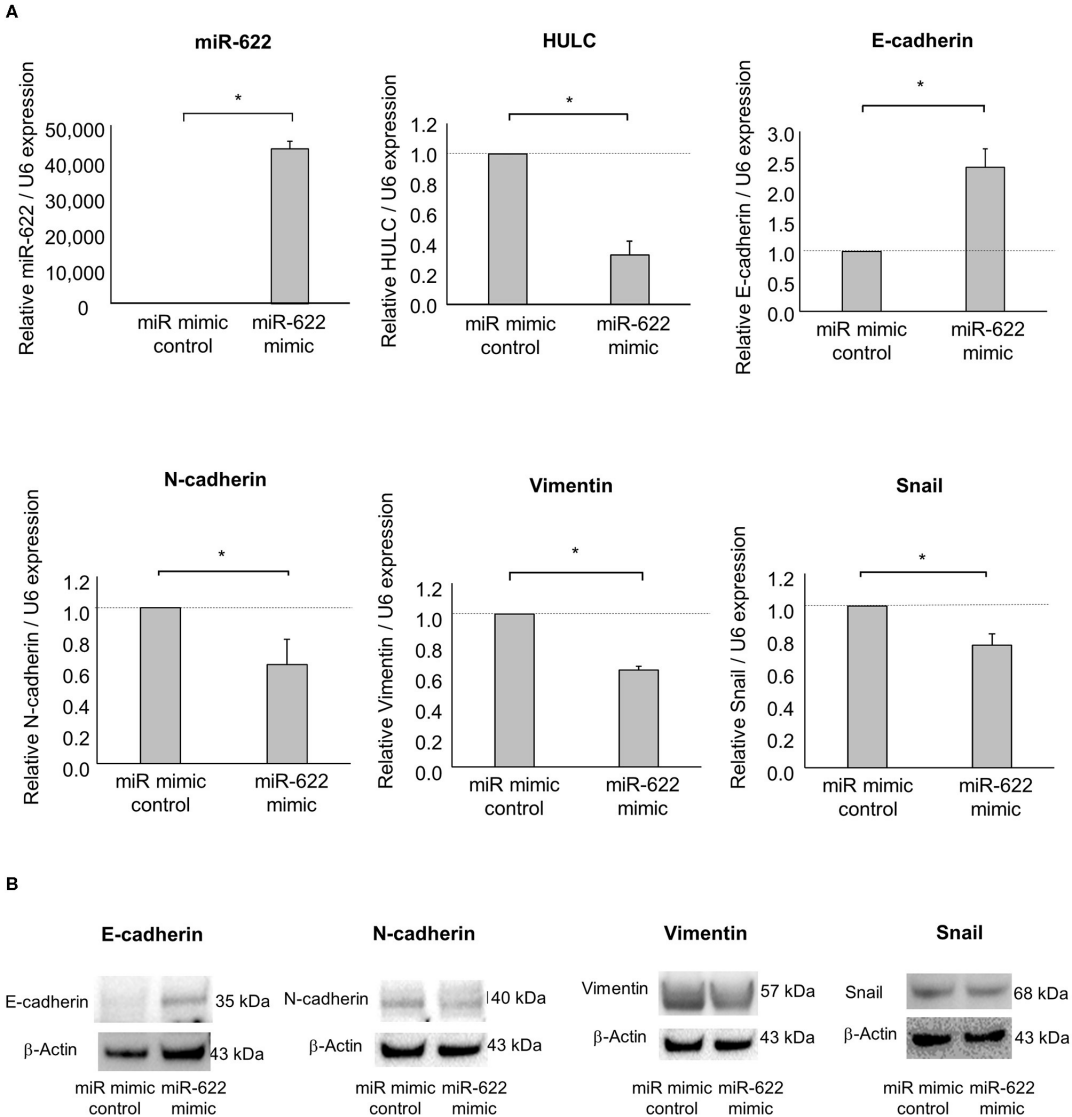
Effect of miR-622 overexpression on EMT in PDAC cells. Panc-1 cells were transfected with 12.5 nM miR-622 or the control mimic. **(A)** After 48 h, RNA was extracted and expression of miR-622, HULC, E-cadherin, N-cadherin, vimentin, and Snail was assessed by qRT-PCR. Bars are the means ± SEM of three independent experiments. *P < 0.05. **(B)** After 72 h, protein was extracted, and western blotting for E-cadherin, N-cadherin, vimentin, and Snail was performed. Protein levels were normalized to that of β-actin.

**Figure 3:**
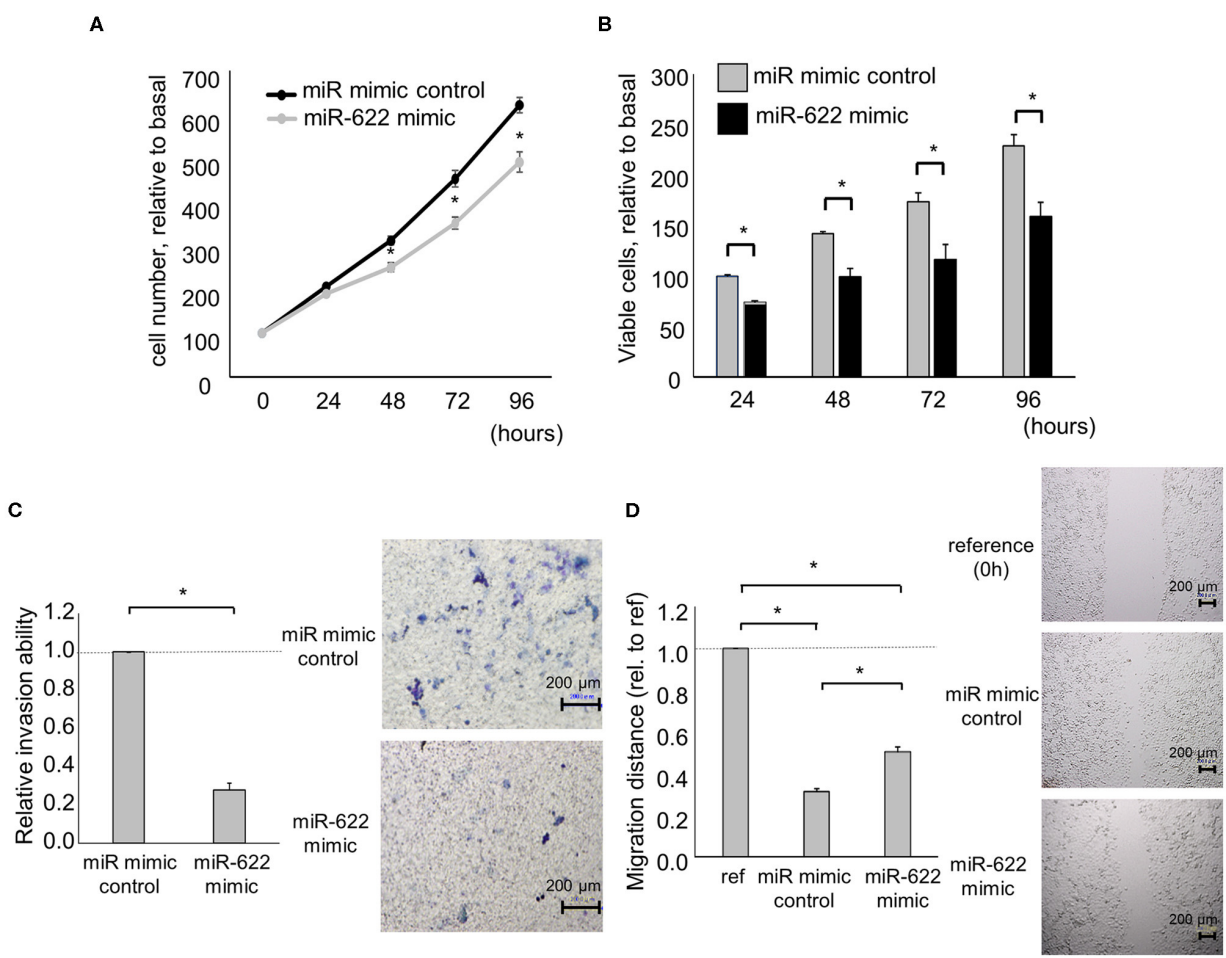
Effect of miR-622 overexpression on the phenotype of PDAC cells. Panc-1 cells were transfected with 12.5 nM miR-622 or the control mimic. **(A,B)** After 24, 48, 72, and 96 h, cell proliferation was assessed by cell counting using trypan blue **(A)** and cell viability was investigated by MTS assay **(B)**. **(C)** After 24 h, cell invasion was examined by Transwell assay using an inverted microscope. **(D)** After 24 h, cell migration was examined by wound healing assay. Reference indicates the image at time zero. Bars are the means ± SEM of three independent experiments. *P < 0.05.

Having identified miR-622 as a HULC-targeting and candidate EMT-suppressive miRNA, we next investigated its role in the EMT pathway via regulation of HULC. To evaluate whether miR-622 suppresses EMT by targeting HULC, we overexpressed miR-622 by using a miR mimic and validated its effect on miR-622 by qRT-PCR. The overexpression of miR-622 down-regulated expression of HULC in PDAC cells. miR-622 overexpression significantly decreased the expression of mRNA and protein of mesenchymal markers N-cadherin and vimentin, and expression of EMT-inducible transcription factor Snail, and increased that of epithelial marker E-cadherin (Figures 2A,B, Figure S1). Moreover, we confirmed that miR-622 overexpression significantly decreased cell proliferation, viability, invasion, and migration of PDAC cells (Figures 3A–D). These observations suggest that the inhibition of HULC, through miR-622 mimic, reduces tumor cell invasion, and migration through suppression of EMT in PDAC cells.

### Inhibition of miR-622 Promotes Cell Invasion and Migration Through Induction of EMT

**Figure 4:**
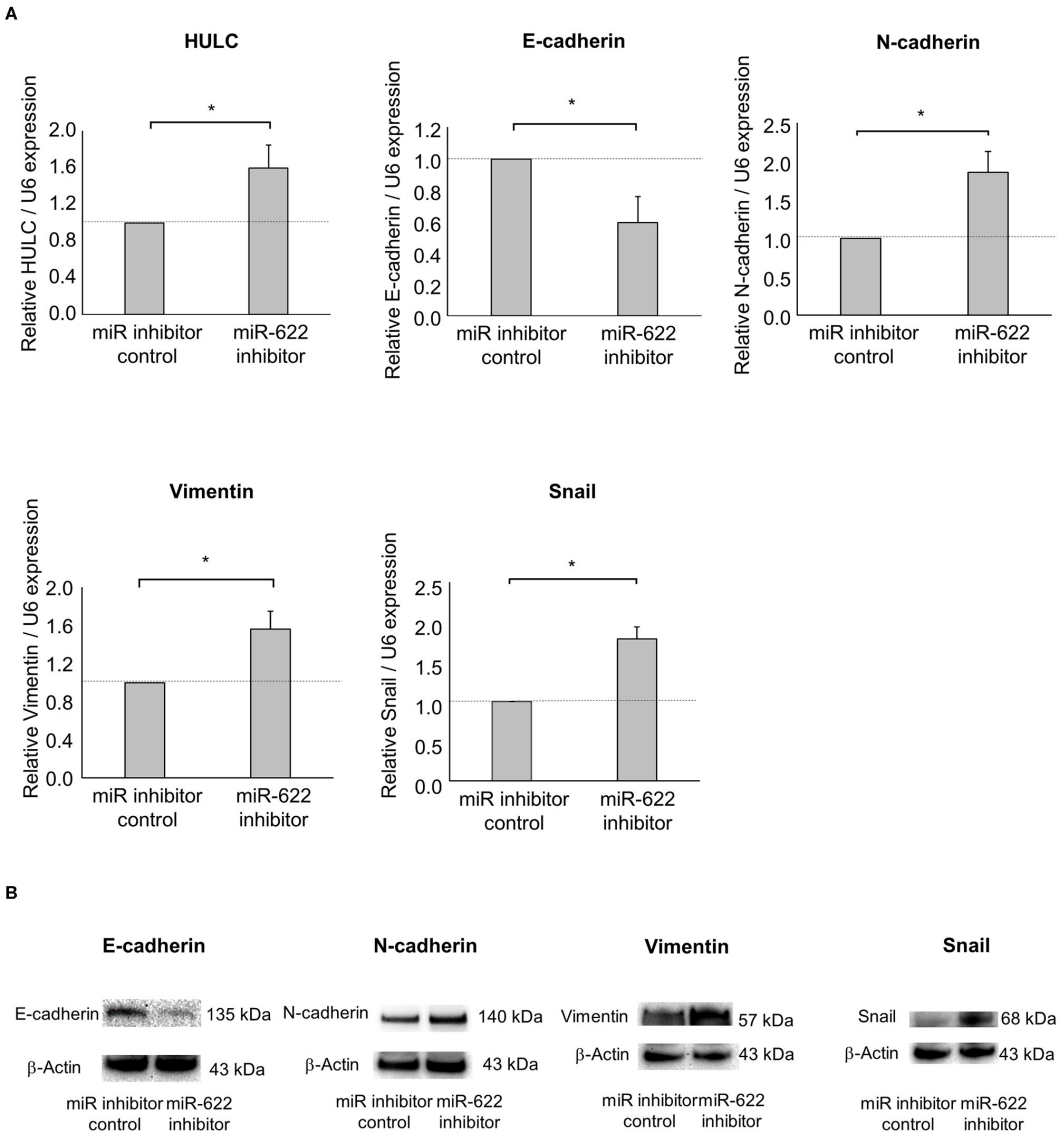
Effect of miR-622 inhibition on EMT in PDAC cells. Panc-1 cells were transfected with 12.5 nM miR-622 or a control inhibitor. **(A)** After 48 h, RNA was extracted and expression of HULC, E-cadherin, N-cadherin, vimentin, and Snail was analyzed by qRT-PCR. Bars are the means ± SEM of three independent experiments. *P < 0.05. **(B)** After 72 h, protein was extracted, and western blotting for E-cadherin, N-adherin, vimentin, and Snail was performed. Protein levels were normalized to that of β-actin.

**Figure 5:**
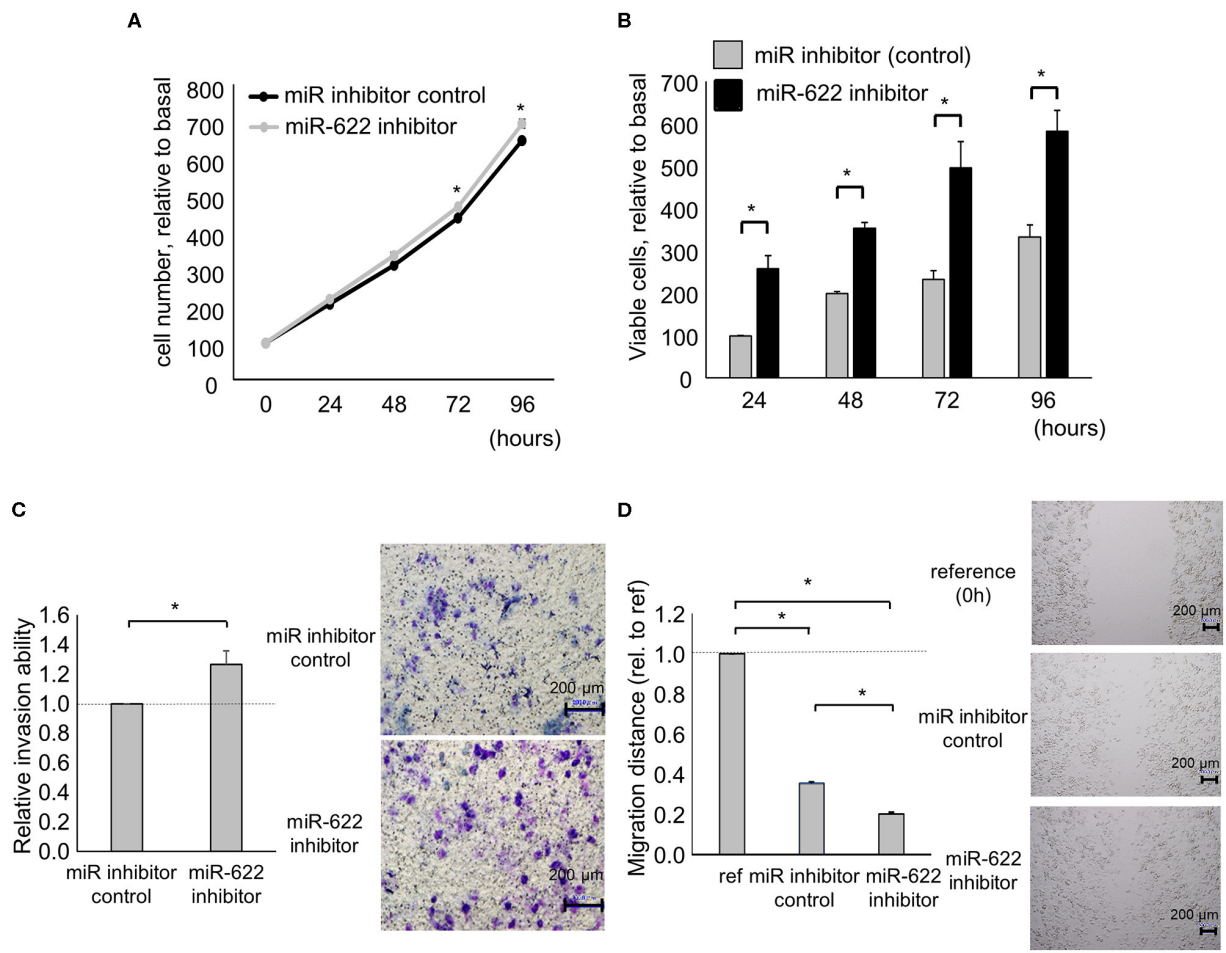
Effect of miR-622 inhibition on the phenotype of PDAC cells. Panc-1 cells were transfected with 12.5 nM miR-622 or a control inhibitor. **(A,B)** After 24, 48, 72, and 96 h, cell proliferation was assessed by cell counting using trypan blue **(A)** and cell viability was investigated by MTS assay **(B)**. **(C)** After 24 h, cell invasion was examined by Transwell assay using an inverted microscope. **(D)** After 24 h, cell migration was examined by wound healing assay. Reference indicates the image at time 0. Bars are the means ± SEM of three independent experiments. *P < 0.05.

Next, to investigate the impact of miR-622 on the regulation of EMT, a miR-622 inhibitor was used to suppress miR-622 activity in PDAC cells. miR-622 inhibition significantly up-regulated HULC expression. Then, we evaluated the effect of miR-622 inhibitor on the EMT-related genes expression in Panc-1 cells. Inhibition of miR-622 significantly up-regulated mRNA expression of N-cadherin, vimentin, and Snail, and down-regulated E-cadherin expression (Figure 4A). Moreover, inhibition of miR-622 could promote the EMT pathway at the protein expression level (Figure 4B). We further assessed tumor-cell phenotypes after miR-622 inhibition. Inhibition of miR-622 significantly increased the proliferation, viability, invasion, and migration of PDAC cells (Figures 5A–D). Altogether, miR-622 may attenuate tumor cell invasion and migration by inhibition of EMT via targeting HULC in PDAC cells.

### miR-622 Transfer by EVs Can Inhibit PDAC Cell Invasion and Migration

**Figure 6:**
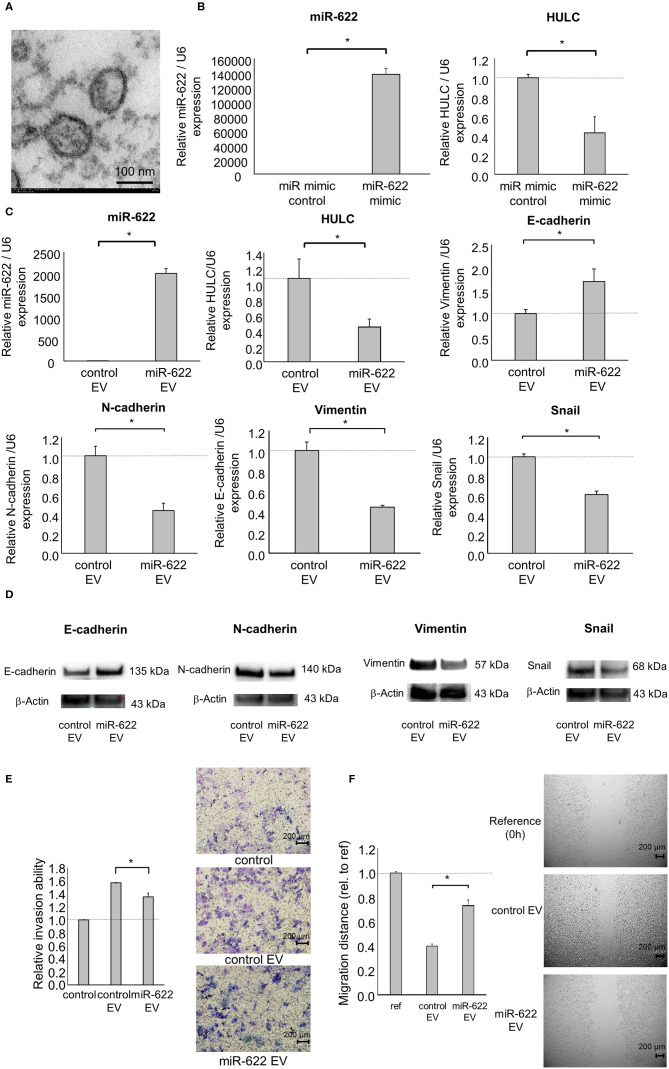
Intercellular miR-622 transfer by EVs during EMT and the phenotype of PDAC cells. Panc-1 cells (1 × 10^6^) were plated in 10 ml of vesicle-depleted medium on 10-cm dishes and were transfected with 12.5 nM miR-622 or the control mimic. After incubation for 72 h, EVs were extracted from the conditioned medium by ultracentrifugation. **(A)** Transmission electron microscopy was performed on EVs isolated from Panc-1 cells. Obtained EVs were composed of a homogeneous population of particles. **(B)** EV RNA was isolated and miR-622 or HULC expression was assessed by qRT-PCR. **(C–F)** EVs were added to recipient Panc-1 cells. **(C)** After 48 h, RNA was extracted from recipient cells and expression of miR-622, HULC, E-cadherin, N-cadherin, vimentin, and Snail was examined by qRT-PCR. RNA expression was normalized to that of U6 and expressed relative to the value of the control. **(D)** After 72 h, protein was extracted, and western blotting for E-cadherin, N-adherin, vimentin, and Snail was performed. Protein levels were normalized to that of β-actin. **(E)** After 24 h, cell invasion was examined by Transwell assay using an inverted microscope. **(F)** After 24 h, cell migration was examined by wound healing assay. Reference indicates the image at time 0. Bars are the means ± SEM of three independent experiments. *P < 0.05.

Similar to mRNAs or proteins, ncRNAs are also transferred by EVs from their donors to recipient cells. We previously reported that EV-mediated transfer of lncRNAs or miRNAs can mediate the phenotypes of neighboring hepatocellular carcinoma (HCC), CCA, and PDAC cells (8, 18, 24–26). Intercellular transfer of miR-30e by CCA cell-derived EVs could inhibit EMT signaling in recipient CCA cells (18). Also, long intergenic ncRNA regulator of reprogramming could be transferred by HCC cell-derived EVs and suppress chemoresistance in recipient HCC cells (26). Based on these findings, we speculated that miR-622 might contribute to the modulation of signaling pathways and behaviors in recipient cells via EV transfer. Thus, we attempted to investigate the ability of EV-encapsulated miR-622 to modulate EMT in recipient PDAC cells. EVs were extracted from PDAC cells by ultracentrifugation and their morphology was validated by electron microscopy. The collected EVs were ~100-nm microstructures with a lipid bilayer membrane (Figure 6A). Next, we collected EVs from donor PDAC cells transfected with miR-622 mimic and confirmed highly elevated expression of miR-622 and down-regulated expression of HULC in those EVs (Figure 6B). Then, we investigated the effect of the transfer of EV-encapsulated miR-622 on the EMT pathway in recipient PDAC cells. Incubation with EVs derived from PDAC cells after transfection with miR-622 mimic significantly increased miR-622 expression compared with incubation with EVs derived from PDAC cells with control mimic, suggesting that miR-622 could be transferred by EVs. Moreover, expression of E-cadherin was increased, and that of HULC, N-cadherin, vimentin, and Snail was decreased, in recipient cells incubated with miR-622-enriched EVs (Figures 6C,D). According to these results, the suppression of EMT by PDAC cell-derived EVs would be mediated by miR-622 through targeting HULC. Furthermore, incubation with miR-622-overexpressing PDAC cell-derived EVs significantly reduced recipient cell invasion and migration (Figures 6E,F). Taken together, these results implicate miR-622 within PDAC cell-derived EVs in suppression of tumor cell invasion and migration by inhibiting the EMT pathway.

## Discussion

miRNAs function as post-transcriptional regulators of gene expression through translational repression or cleavage of mRNA, mediated by recognition of complementary sequences of target mRNAs (27, 28). Although miRNAs can act as oncogenes or onco-suppressor genes (29), miR-622 has been reported to work as a tumor suppressor in several cancers. In HCC, miR-622 directly targets the 3′ untranslated region of CXC chemokine receptor 4 (CXCR4) and inhibits tumorigenesis via suppression of CXCR4 expression (30). miR-622 was also reported to inhibit metastasis through regulating the EMT pathway by suppression of hypoxia-inducible factor-1α in lung cancer (31). Yes-associated protein 1 (YAP1) was identified as an oncogene in several cancers, including HCC, gastric adenocarcinoma, and non-small cell lung cancer. miR-622 can directly target YAP1 and inhibit cell proliferation of glioma cells by downregulating the expression of YAP1 (32). However, there is no literature on the roles of miR-622 in PDAC development. Our study revealed that miR-622 was down-regulated by TGF-β1 and could attenuate tumor migration and invasion via suppression of EMT by targeting HULC. This is the first report that miR-622 functions as an onco-suppressor gene in human PDAC. These observations may lead to the discovery of novel therapeutic approaches. The limitation of our study is that it is unclear whether TGF-β1 is acting through the canonical or non-canonical pathway in miR-622 expression. To investigate the issue, future studies will be needed to evaluate the expression of phosphor-SMAD2/3 and phosphor-ERK. Another limitation would be that there is no correspondence between the high expression of miR-622 and the downregulation of HULC, in particular when the cells are transfected with miRNA mimic. HULC would be one of the important miR-622 targets as well as the EMT inducer in PDAC cells (8). However, future studies focusing on the investigation of miR-622 target genes, which are more specifically modulated and contribute to the regulation of EMT, will be required using several PDAC cell lines.

LncRNAs have been reported to contribute to many biological processes by diverse mechanisms in cancers (5). The interrelationship between two classes of ncRNA, miRNAs, and lncRNAs, has been known to affect the epigenetic regulation in several diseases. Some investigators have shown that several lncRNAs are directly or indirectly targeted by miRNAs to repress the expression of lncRNAs (33). miR-34a acts as a tumor suppressor gene by inhibiting prostate cancer cell growth. miR-34a binds directly to the HOX transcript antisense RNA (HOTAIR) and represses the expression of HOTAIR (34). miR-29 can regulate the expression of MEG3 in methylation-dependent and -independent manners, and contribute to HCC cell growth (35). Similar to these findings, we present new insights into the miRNA–lncRNA interaction by showing that miR-622 directly targets HULC and attenuates PDAC cell invasion and migration via regulation of HULC. In our present and previous studies, both miR-133b and miR-622 were decreased during TGF-β induced EMT, and both could directly target HULC (8). Single inhibition of either miR-133b or miR-622 produced similar results. These data suggested potential compensatory mechanisms with inhibition of just one miRNA. Because an mRNA can be targeted by multiple miRNAs, the limitation of these two studies would be that we did not elucidate when miR-622 can predominantly work rather than miR-133b and how those two miRNAs works compensatory. The detailed mechanisms should be investigated to fully understand the contributions of the miR-622–HULC interaction in the regulation of EMT in the future studies. However, the present study reveals previously unrecognized roles of miR-622 in PDAC development.

EVs have been implicated in invasion and metastasis in several cancers. While some EV contents have been reported to induce tumor invasion and metastasis, other factors have also been recognized to inhibit cancer development (13, 36). Breast cancer-derived EVs encapsulate miR-122 and the intercellular transfer of miR-122 by EVs could promote breast tumor metastasis, whereas EVs derived from patients with non-metastatic primary melanomas suppress lung metastasis (37, 38). Moreover, cancer cell-derived EVs are thought to play a role in metastatic organotropism of breast and pancreatic cancers via integrin expression in EVs (39). In our present and previous studies, negative control EVs derived from PDAC cells increased cell invasion and migration in receiving cells (8). This particular aspect of these data suggested that additional EV contents, such as oncogenic mRNAs, ncRNAs, or proteins, could be involved in inducing EMT and tumor cell development. On the other hand, EV miR-622 could attenuate the EMT, invasion, and migration in receiving cells. Because most exRNAs would be encapsulated within EVs, EV contents other than miR-622 might also have been transferred to recipient PDAC cells and may have affected EMT signaling in the present study. However, miR-622 is confirmed to be within PDAC cell-derived EVs, and miR-622-overexpressing EVs could deliver miR-622 and suppress recipient cell invasion and migration through inhibiting EMT, suggesting that extracellular miR-622 would be contained within the EVs and could be one of the most important factors for regulation of EMT in PDAC. This is the first report that miR-622 is a miRNA that can be encapsulated and carried by cancer cell-derived EVs. Although further studies are needed to investigate the roles of miR-622 and HULC in PDAC development, our findings indicate that the interaction between EV-miR-622 and HULC mediates EMT, as well as invasion and migration, in PDAC cells.

We demonstrated the role of lncRNA-miRNA signaling in tumor invasion and migration in PDAC cells, and revealed the contribution of EV transfer of ncRNAs to PDAC development. These observations provide new insights into cancer cell invasion and metastasis and identify EV-encapsulated ncRNAs as novel therapeutic targets for human PDAC.

## Data Availability Statement

The datasets generated for this study can be found in the miRNA microarray data can be accessed in the NCBI GEO Database (https://www.ncbi.nlm.nih.gov/geo/query/acc.cgi) under accession no. GSE121369.

## Ethics Statement

This study was approved by the Institutional Review Board of Asahikawa Medical University.

## Author Contributions

KT designed the project, organized data, and wrote the manuscript. KT, KK, and YO performed the experiments and analyzed data. HI, KY, and YK reviewed the data and manuscript. SF conducted experiments, data analysis, and manuscript preparation. All authors contributed to the article and approved the submitted version.

## Conflict of Interest

The authors declare that the research was conducted in the absence of any commercial or financial relationships that could be construed as a potential conflict of interest.

## Supplementary Material

Figure S1 Effect of miR-622 overexpression on EMT in PDAC cells. MIA PaCa-2 cells were transfected with 12.5 nM miR-622 or the control mimic. After 48 h, RNA was extracted and expression of miR-622, HULC, E-cadherin, N-cadherin, vimentin, and Snail was analyzed by qRT-PCR. Bars are the means ± SEM of three independent experiments. ^*^*P* < 0.05.
