# Supplementary figures and images for: The Interaction Between Long Non-coding RNA HULC and MicroRNA-622 via Transfer by Extracellular Vesicles Regulates Cell Invasion and Migration in Human Pancreatic Cancer
Source: Front Oncol. 2020 Jun 23;10:1013. doi: 10.3389/fonc.2020.01013 (PMC7324724; doi:10.3389/fonc.2020.01013)

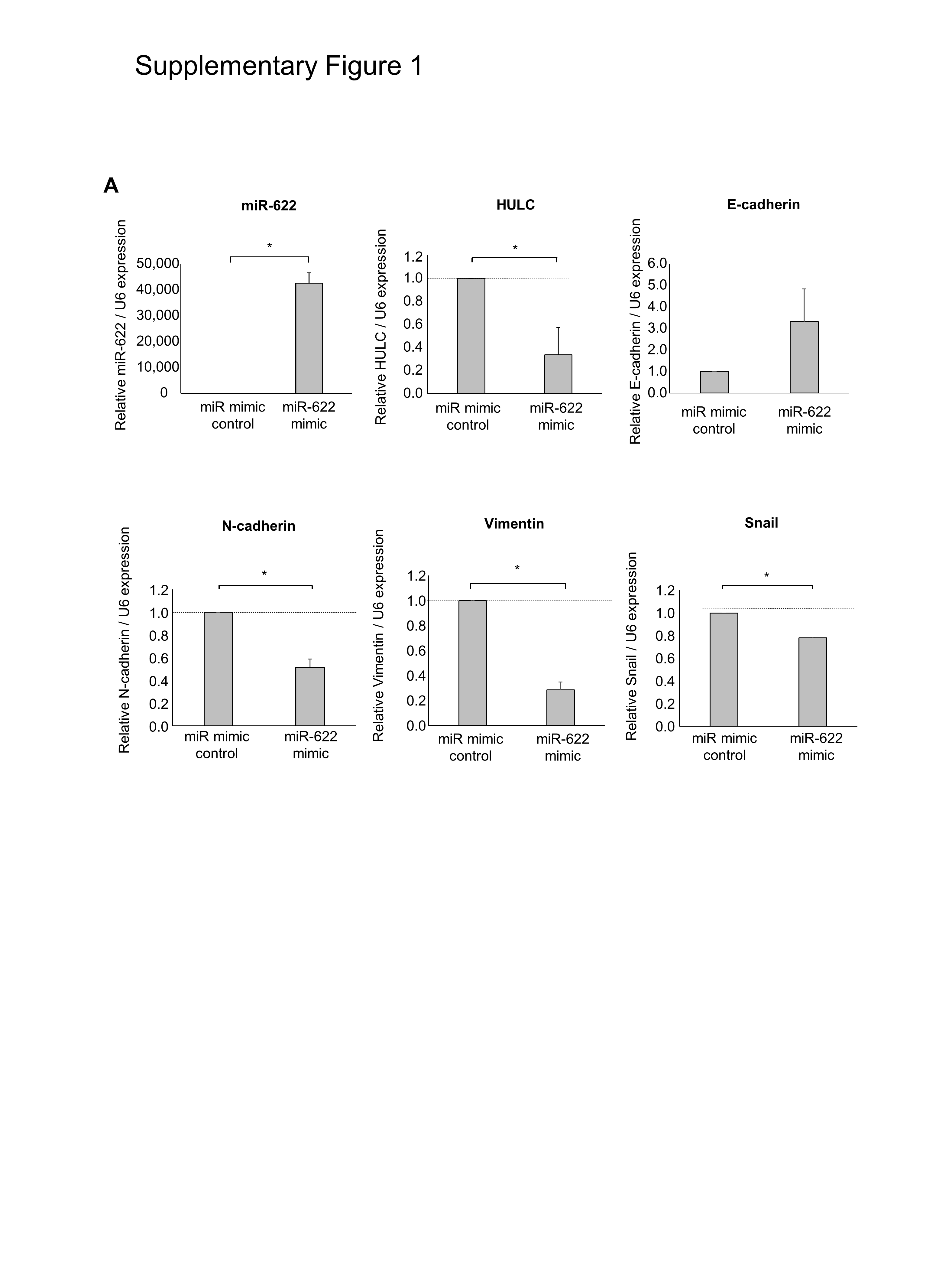

Supplement: Figure S1 — Effect of miR-622 overexpression on EMT in PDAC cells. MIA PaCa-2 cells were transfected with 12.5 nM miR-622 or the control mimic. After 48 h, RNA was extracted and expression of miR-622, HULC, E-cadherin, N-cadherin, vimentin, and Snail was analyzed by qRT-PCR. Bars are the means ± SEM of three independent experiments. *P < 0.05. [file Image_1.TIF]
